# B‐lymphoblastic leukemia with Burkitt‐like morphology and aberrant myeloperoxidase expression: A diagnostic conundrum

**DOI:** 10.1002/jha2.578

**Published:** 2022-09-22

**Authors:** Karen A. Nahmod, Wei Wang, Karan Saluja, Zhenya Tang, L. Jeffrey Medeiros, Beenu Thakral

**Affiliations:** ^1^ Department of Hematopathology The University of Texas MD Anderson Cancer Center Houston Texas USA; ^2^ Department of Pathology and Laboratory Medicine, McGovern Medical School The University of Texas Health Science Center at Houston Houston Texas USA

**Keywords:** B‐lymphoblastic leukemia, Burkitt‐like, flow cytometry, immunohistochemistry, myeloperoxidase

1

A 48‐year‐old woman was diagnosed with B‐acute lymphoblastic leukemia (B‐ALL) in 1996 and was treated with chemotherapy and an allogeneic sex mismatched unrelated donor stem cell transplant (SCT) in 1997 at another institution. She presented to our hospital in January 2020 with pancytopenia. Bone marrow (BM) biopsy (Figure 1) showed hypercellular (95%) BM with sheet of blasts (A, hematoxylin and eosin, 10×). Aspirate smears showed 80% blasts that were large with dispersed chromatin, inconspicuous nucleoli, and basophilic cytoplasm with numerous vacuoles reminiscent of Burkitt‐like morphology (B, Wright and Giemsa, 50×). Flow cytometry (FC) immunophenotyping showed B‐lymphoblasts positive for CD19, CD20 (subset), CD22, CD34, CD38 (decreased), CD79a, HLA‐DR, and TdT (C and D) with an aberrant myeloperoxidase (MPO) expression (E) that was dimmer in blasts (red) compared with background granulocytes (gray). Blasts were negative for myeloid and/or monocytic markers including CD11c, CD13, CD14, CD33, CD64, CD117, and lysozyme. Immunohistochemistry highlighted blasts positive for Pax5 (F, 20×) and MPO with a granular cytoplasmic staining pattern (G, 20×). Cytochemistry showed 35% of blasts positive for MPO (H, 500×) and negative for butyrate esterase. Fluorescence in situ hybridization analysis was negative for *KMT2A/MLL* rearrangement and *BCR::ABL* fusion. Conventional cytogenetic analysis showed a near‐triploid female karyotype with additional structural abnormalities: 68∼72 <2n>, XX, +1, +1, +2, +3, +4, +5, +6, +del(6)(q16), +8, +10, +10, +11, +11, +12, +13, +18, +18, +19, +21, +21, +21, +22, +1 ∼2mar[cp19]//46,XY [1]. A single metaphase with a male karyotype represent donor cell (prior history of sex mismatched SCT). These findings support the diagnosis of B‐ALL.

**FIGURE 1:**
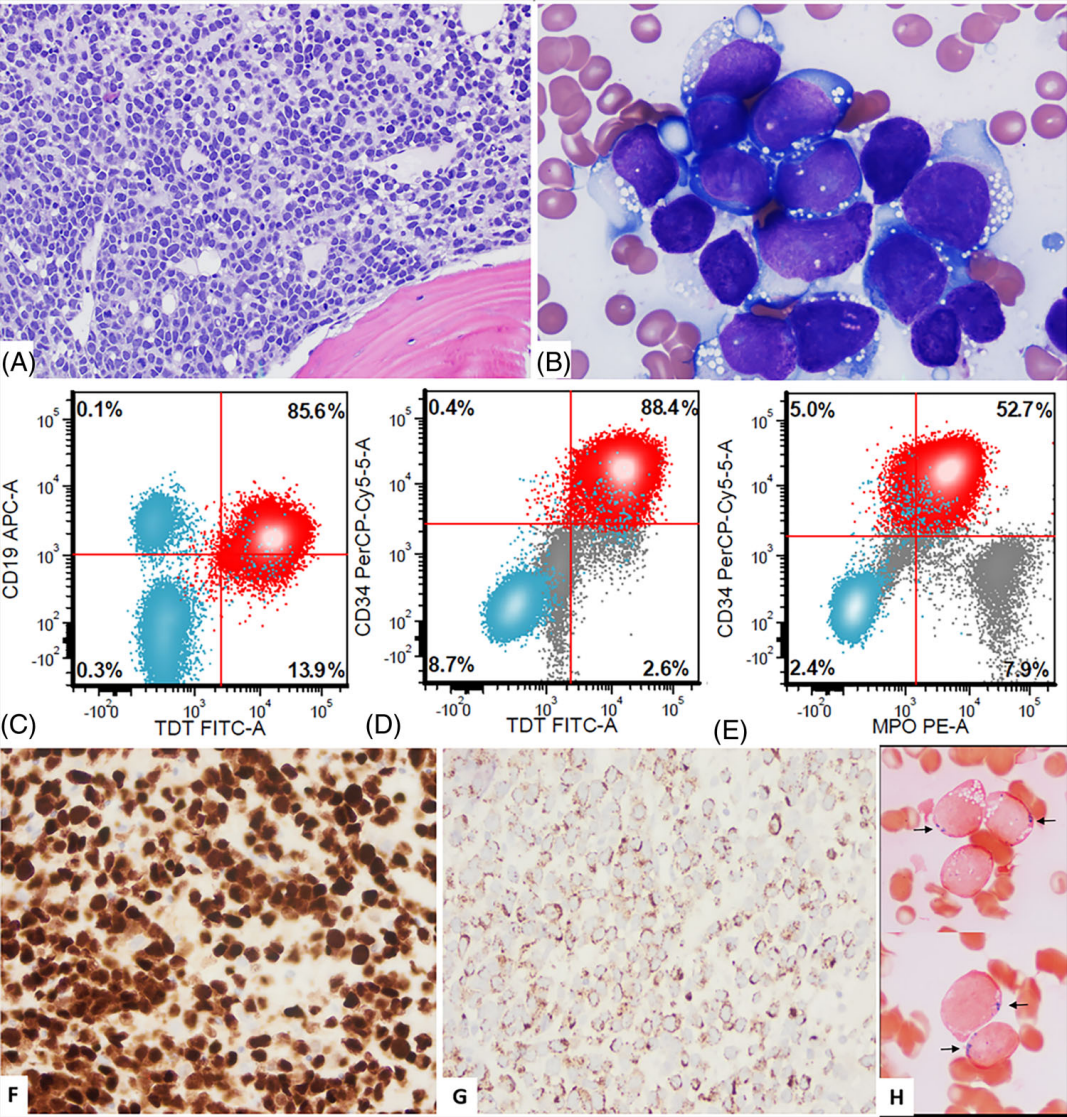
(A–H) B‐lymphoblastic leukemia with Burkitt‐like morphology and aberrant myeloperoxidase (MPO) expression.

This post‐transplant case of relapsed B‐ALL had interesting features as the blasts had Burkitt‐like morphology but were negative for CD10 and positive for the immature markers CD34 and TdT. In addition, the blasts showed an aberrant MPO expression (as defined by ≥3% positivity in blasts by cytochemistry by World Health Organization [WHO] or ≥10% by FC as defined by the European Group for the Immunological Classification of Leukemia) bringing the possibility of mixed‐phenotype acute leukemia (MPAL), B/myeloid into the differential diagnosis.

Cases of typical B‐ALL with high expression of MPO (>20%) in blasts are rare with a handful of cases reported in the literature [1, 2, 3, 4]. In addition, to the peculiar Burkitt‐like cytomorphologic features, this case also illustrates the diagnostic challenge of using “only” MPO expression as a sole myeloid defining marker. Similar to the case we report, many of these cases in literature show Burkitt‐like morphology, express dim MPO with a characteristic granular staining pattern, show no other myelomonocytic markers and respond well to B‐ALL type therapy [1, 2, 3, 4]. The literature review, cytogenetics results and per WHO 2017 revised classification [5] guidelines, “if no additional myeloid or monocytic markers besides MPO are expressed, it is best to classify these cases as B‐ALL instead of MPAL” as seen in our case helped us in achieving the correct diagnosis of B‐ALL. Our patient received mini‐hyperfractionated cyclophosphamide, vincristine, and dexamethasone with rituximab, inotuzumab, and blinatumomab for relapsed B‐ALL, is currently on POMP (prednisone, vincristine, methotrexate, and mercaptopurine) maintenance therapy and remains negative for measurable residual disease B‐ALL after 1.5 years. Distinguishing B‐ALL from MPAL is of paramount importance because of the differences in therapy and prognosis.

## AUTHOR CONTRIBUTIONS

Karen A. Nahmod and Beenu Thakral wrote the text. Beenu Thakral took images and signed out the relapsed B‐ALL bone marrow. Wei Wang analyzed the flow cytometry data. Karan Saluja and L. Jeffrey Medeiros follow‐up of the case. L. Jeffrey Medeiros analyzed fluorescence in situ hybridization (FISH) data. Zhenya Tang analyzed cytogenetic results. All the co‐authors have revised the article critically and approved the final version.

## CONFLICT OF INTEREST

The authors declare they have no conflicts of interest.

## FUNDING INFORMATION

The authors received no specific funding for this work.

## PATIENT CONSENT STATEMENT

The patient signed the institution‐approved standard consent for clinical diagnostic testing.
